# Efficient Conversion of Mushroom and Sawdust Residues in *Protaetia brevitarsis* Biosystem: Characterization of Humic Acid and Bacterial Communities

**DOI:** 10.3390/insects16090893

**Published:** 2025-08-26

**Authors:** Abdelaziz Mansour, Junbeom Lee, Taeho Jeong, Mohamed Mannaa, Sun Young Kim, Jeong-Hun Song, Young-Su Seo, Dae-Weon Lee

**Affiliations:** 1 Department of Integrated Biological Science, Pusan National University, Busan 46241, Republic of Korea; abdelaziz.mansour@agr.cu.edu.eg (A.M.); mannaa@cu.edu.eg (M.M.); 2 Department of Economic Entomology and Pesticides, Faculty of Agriculture, Cairo University, Giza 12613, Egypt; 3 Metabolomics Research Center for Functional Materials, Kyungsung University, Busan 48434, Republic of Korea; 4 Department of Plant Pathology, Faculty of Agriculture, Cairo University, Giza 12613, Egypt; 5 Industrial Insect and Sericulture Division, National Institute of Agricultural Sciences, Wanju 55365, Republic of Koreajeonghuns@korea.kr (J.-H.S.); 6 Department of SmartBio, Kyungsung University, Busan 48434, Republic of Korea

**Keywords:** organic waste, agricultural residues, insect frass, bio-fertilizer, microbes

## Abstract

The accumulation of agricultural residues presents an environmental challenge. PBLs have emerged as effective agents for biodegrading such biomass, producing frass rich in HA with low phytotoxicity, positioning it as a potential biofertilizer. However, the influence of PBL bioconversion on HA yields and microbial communities across different substrates remains underexplored. In this research, PBL is fed on two *Pleurotus* SMSs and oak sawdust. The resulting frass was characterized and showed low phytotoxicity based on seed germination and plant growth. The extracted HA quantity and quality were significantly higher in frass than diet samples. Microbial profiling using 16S rRNA gene high-throughput sequencing revealed the enrichment of potential PGP genera, including *Pseudoxanthomonas*, *Cellulomonas*, Flavobacterium, and *Mucilaginibacter*. In addition, the actino-genera *Cellulomonas*, *Demequina*, *Xylanimicrobium*, *Mycolicibacter*, *Nakamurella*, and *Glutamicibacter* were positively correlated with HA content and quality parameters. This study highlights the potential of PBL systems in waste valorization and biofertilizer production as a novel approach for sustainable agriculture.

## Simple Summary

*Protaetia brevitarsis* larva (PBL) can feed on a wide range of organic waste substrates. Despite its documented ability to convert agricultural byproducts, its efficiency to convert *Pleurotus* spent mushroom substrates (SMSs) and sawdust to frass-fertilizer remains unclear. In this study, to explore the efficiency of PBL to convert SMSs and sawdust substrates to frass-fertilizer, the phytotoxicity of PBL frass was assayed based on tomato germination and growth. Subsequently, the impact of PBL-based conversion on humic acid (HA) quantity and quality was analyzed by using UV-spectrophotometer analysis. The bacterial composition in feeding substrates and frass samples was investigated using 16S rRNA amplicon sequencing. The findings of this study revealed that PBL can efficiently convert SMSs and sawdust substrates to frass fertilizer with a high HA content and plant growth-promoting (PGP) microbes with low phytotoxicity.

## 1. Introduction

Agricultural waste stream, which is a byproduct of farming activities, is considered a buried treasure due to its potential benefits and applications. These byproducts include, for example but not limited to, livestock manure, crop straw, woody sawdust, and mushroom residues [1]. The spent mushroom substrates (SMSs) are rich in lignocellulosic biomass (LCB) and generated after mushroom cultivation. *Pleurotus* is one of the main edible mushroom genera that highly contribute to the global mushroom production and, consequently, result in a massive amount of residual substrates [2]. One of the main species belonging to this group is the oyster mushroom, *Pleurotus ostreatus*, which is commonly cultivated on decaying woodchips and distributed worldwide because of its favorable taste and high nutritional and medicinal values [3,4]. *Pleurotus eryngii*, or the king oyster mushroom, has a unique texture and flavor, making it a very popular dish in several world regions, predominately in Europe, North America, Asia, and the Middle East [5,6]. Oak sawdust, which is a lignocellulosic material, is a forest byproduct derived from wood processing worldwide. In Korea, fermented oak sawdust is commonly used as a feeding substrate for PBL-rearing in many farms [7].

The accumulation of SMS and sawdust substrates causes a serious environmental issue due to improper disposal practices, such as landfilling or incineration. Much research has focused on employing biotechnological tools to utilize SMSs into value-added products, including biofuel [8], bioremediation agents [9], and biofertilizer [10]. The conversion of SMSs into biofertilizer and soil amendment are noted to be performed using soil invertebrates, such as earthworms and insect larvae [11,12]. In comparison to earthworms, insects have a higher efficiency in degrading LCB materials in a short time, and the resulting frass after larval feeding was found to have a high HA amount [13]. HA is a complex chemical compound that contains various functional humic substances, and it was found to have several applications in industry, medicine, the environment, and agriculture [14,15]. In agriculture, HA is reported to have many benefits for plant growth, including increasing the capacity of water holding [13], nutrition absorption [16], soil pH [17], and remediation of heavy metals [18,19]. The commercial HA is mainly produced based on non-renewable sources, like coal and peat, and that creates critical needs to find alternative renewable sources [17]. Cutting-edge research now focuses on the valorization of renewable HA sources, including vermicompost, biochar, and insect frass [13,20,21]. The insect frass source has been considered a promising and emerging sustainable agricultural solution that enhances productivity while mitigating environmental impacts [22].

The larvae of *Protaetia brevitarsis* (PBL) can feed on a wide range of waste materials and was shown to have high ability to convert plant residues into protein biomass and frass-based biofertilizer [23,24]. PBL was found to efficiently degrade LCB waste and produce frass with low phytotoxicity, high HA content, and beneficial microbes [13,25]. The PBL-mediated degradation is apparently passing, through grinding LCB substrates using strong mouthparts, dissolving crashed particles in the alkaline midgut, and fermentation occurring in the hindgut [26]. The genome annotation indicates that PBL could not completely perform the LCB decomposition but can with the help of its microbial symbionts and related enzymes. In addition, the presence of PGP microbes that promote plant growth and disease resistance was found in PBL frass [25,27]. However, the impact of PBL-based conversion on the HA content and bacterial composition in SMS and sawdust substrates is still not clear. Therefore, the main goal of this study was to feed PBL with these substrates and characterize the HA extracted from the diet and resulting frass. In parallel, the aim was to figure out the phytotoxic effects of PBL frass on seed germination and plant growth. As we aimed to understand the influence of PBL conversion on microbial composition, the bacterial communities in diet and frass samples were investigated. A characterization of HA contents and bacterial communities in PBL frass will ensure that frass delivers maximum agronomic and ecological benefits, supporting both crop productivity and soil health in a sustainable manner.

## 2. Materials and Methods

### 2.1. Insect Rearing and Frass Collection

The insects and diet substrates of this study were provided by the National Institute of Agricultural Sciences, Rural Development Administration, Republic of Korea (www.rda.go.kr). The PBL was raised in a growth chamber at a temperature of 27 ± 1 °C and with 60–70% relative humidity. The third instar larvae were fed at ratio of 5 g of moisture diet to 1 g of fresh larval biomass, based on the three different diet substrates: (A) king oyster mushroom residues (*Pleurotus eryngii*), (B) fermented residues of oyster mushroom (*Pleurotus ostreatus*), or (C) fermented oak sawdust. The treatments were performed in round plastic boxes (8 cm × 10 cm) with three replicates for each diet. After one month of feeding, fresh PBL frass (granulated manure and feeding residues) was collected and divided into two portions: one kept at −20 °C, and the other one was freeze-dried for subsequent analysis.

### 2.2. Phytotoxicity Assessment

To evaluate the phytotoxicity of PBL frass after feeding on diet substrates, the germination index (GI %) of tomato seeds was investigated. The tomato used in this experiment, Hybrid Cherry Tomato (*Solanum lycopersicum* L.), Berry King F1, was purchased from Asia seed Co., Ltd., Seoul, Republic of Korea (http://asiaseed.net/). The frass extracts were prepared according to the methods mentioned in [13], with some modifications. Lyophilized frass samples were dissolved in distilled water at 1:10 (mass/volume). The mixtures were then shaken for 2 h at 30 °C and centrifuged at 10,000× *g* for 15 min, and the pH value was recorded before and after the shaking incubation. The supernatants were then 10-fold diluted using distilled water, transferred to a new tube, and filtered through a 0.22 µm micro-filter (Merck Millipore, Darmstadt, Germany). Seed germination tests were conducted with frass extracts and tomato seeds. Four-layered filter paper beds were placed into a sterile culture dish (9 cm diameter) and soaked in 10 mL of frass extract, and ten tomato seeds were then distributed on each filter paper bed. Distilled water was used as a control, and the experiment was set using three replicates for the treatment and control. After incubation at 25 °C in dark conditions for 4 days, the GI index of each sample was calculated using the following equation:

GI % = (ANT × ALT)/(ANC × ALC)

ANT and ALT are the average number and root lengths of germinated seeds in the treatment, respectively, while ANC and ALC are the average number and root lengths of germinated seeds in the control, respectively.

### 2.3. Plant Growth Assay

This assay was also performed to evaluate the phytotoxic effects of PBL frass on plant growth parameters. Tomato seeds were germinated by soaking ten seeds in 10 mL of distilled water on four-layered filter paper beds, which were placed into a 9 cm sterile culture dish. After incubation at 25 °C in dark conditions for 4 days, the best radicles were selected and transplanted into a plastic pot filled with the cultivation soil (Hanareum Sangto, Shinsung Mineral Co., Ltd., Seongnam, Republic of Korea), with three radicles/pot, adding an adequate amount of tap water, and stored at 25 °C under 12:12 h light:dark conditions for 7 days. The tomato seedlings were then individually transferred to a new soil pot and incubated under the same conditions for 30 days. Frass extracts were prepared as mentioned above, and then, 5 mL of frass extract was mixed with 45 mL of distilled water (50 mL in total) and administered to the tomato seedlings twice, with the first after two weeks of seedling transplantation and the second time after a week of the first treatment. After 30 days of incubation, the growth parameters were measured, including the fresh weight and plant length, using ImageJ software. Images were captured using a Nikon camera (D5100, Nikon, Tokyo, Japan) equipped with a VR-lens (18–55 mm). This experiment was set using 5 replicates, and distilled water was used as a control.

### 2.4. Humic Acid Extraction

The chemical separation of HA depends on the water solubility of humic substances under various pH conditions. The HA was extracted from the PBL diet and frass samples according to the method of [13], with a slight modification. In brief, two grams of lyophilized samples were dissolved in a 50 mL confocal tube with 20 mL of a 0.1 M NaOH solution. After shaking for 24 h at 30 °C, the mixture was centrifuged at 10,000 rpm for 15 min, and the dark pellet was decanted; the supernatant containing humic acids was then transferred to a new tube and acidified with 3 M HCl to modify the pH until it was 1.0–1.5 to allow for the separation of HA fractions. The acidified supernatant was left at room temperature for 24 h and then centrifuged again at 10,000 rpm for 15 min. After discarding the supernatant, the HA pellet was suspended in 10 mL of 0.1 M NaOH and dialyzed using a Spectra/Por^®^ dialysis membrane with a 500–1000 Da molecular weight cut-off (Spectrum Laboratories, Inc., Rancho Dominguez, CA, USA). After centrifugation at 10,000 rpm for 15 min, the HA gel was transferred to a pre-weighed 50 mL Falcon tube for freeze-drying and weighing on a microbalance. The HA powder was then kept at −80 °C until the next analysis.

### 2.5. UV-Spectrophotometer

For UV spectrophotometer analysis, the standard HA was dissolved in a 0.1 M NaOH solvent. From this solution, serial standard solutions involving 0.01–0.5 mg/mL HA were prepared using 0.1 M NaOH for calibration. The extracted HA was dissolved in 20 mL of 0.1 M NaOH solvent; then, 2 mL was taken and diluted (1:1) using 0.1 M NaOH for the UV measurements. The absorbance of humic substances was measured using a UV-spectrophotometer (UV-1800, Shimadzu Co., Kyoto, Japan) in the mixture of 0.1 M NaOH by transferring the mixture into a 10 mm disposable cuvette and recording its absorbance at 280, 465, and 665 nm. The E4/6 ratios were determined by taking the ratio of absorbance at 465 and 665 nm of the UV-spectrophotometer reads. ΔlogK values were calculated as follows: log E465 − log E665.

### 2.6. DNA Extraction and Sequencing

Metagenomic DNA extraction was performed using a PowerSoil^®^ DNA Isolation Kit (Qiagen, Hilden, Germany; previously Mo Bio Laboratories). Diet samples were suspended in the dialysis solution of PowerBead tubes, and then, the steps of the manufacturer’s protocol were followed. Frass samples need to be processed before DNA extraction. They were suspended in 15 mL of sterile distilled water. Under sterilized conditions, samples were vortexed for 1–2 min and filtered through a qualitative filter paper (ADVANTEC, Dublin, CA, USA, grade 2, 110 mm). The filtrate was centrifuged at 5000× *g* for 20 min to remove excess water from the suspension. Pellets of each sample were suspended in the dialysis solution of PowerBead tubes, and then, the steps of the manufacturer’s protocol were followed. The quality and concentration of the obtained metagenomic DNA was evaluated using agarose gel electrophoresis and a NanoDrop2000 spectrophotometer (Thermo Fisher Scientific, Wilmington, NC, USA). Samples were then stored in a 10 mM Tris-HCl solution at −20 °C for subsequent analyses. Metagenomic analysis was conducted according to the NGS library preparation workflow using the hypervariable V3 and V4 regions of the 16S rRNA, which were sequenced using an Illumina^®^ MiSeq^®^ platform at Macrogen (Seoul, Republic of Korea), using the following primers: Bakt_341F (CCTACGGGNGGCWGCAG) and Bakt_805R (GACT ACHVGGGTATCTAATCC) [28].

### 2.7. Bioinformatic Analysis

The resulting raw paired-end sequences were then assembled using Fast Length Adjustment of Short Reads (FLASH, Version 1.2.11) [29]. After trimming adaptors and short read sequences, the purified merged sequences were analyzed using the Divisive Amplicon Denoising Algorithm (DADA2) tool [30]. Briefly, DADA2 was employed to remove sequences containing ambiguous bases (Ns) or more than two expected errors and to trim the first 20 nucleotides, as well as the last 10 nucleotides (for forward reads), or between 10 and 50 nucleotides (for reverse reads). Joined reads were quality trimmed (Phred score < 20), and short reads (<250 bp) were discarded. Chimeric sequences were eliminated from the denoised output using *isBimeraDenovo* in the DADA2 R package. The purified sequence reads were then clustered and annotated into Amplicon Sequence Variants (ASVs) using UCLUST algorithms, with a 97% cut-off based on the Greengenes database for bacterial taxonomic assignments. The microbiome analyses were then performed following the pipeline of Quantitative Insights into Microbial Ecology version 2 (QIIME2), to implement alpha and beta diversity statistics and taxonomic assignment of the detected ASVs [31]. Then, species classification and subsequent analysis were performed using the BLAST tool in the National Center for Biotechnology Information (NCBI, 16S_20230616) database. The obtained sequences were submitted to NCBI as a sequence read archive (SRA) under the following BioProject ID: PRJNA1071350.

### 2.8. Statistical Analysis

The statistical analyses of phytotoxicity assays, plant growth parameters, HA quantity and quality, and alpha diversity data were performed using IBM SPSS Statistics (version 21) with an analysis of variance (ANOVA), and a *p*-value < 0.05, as per an LSD post hoc test, was considered statistically significant. The statistical differences between the two groups (diet and frass) in HA and alpha diversity data were determined using an independent two-tailed *t*-test. All data were analyzed after confirming their normal distribution using a Shapiro–normality test (*p*-value > 0.05, https://www.statskingdom.com/shapiro-wilk-test-calculator.html, accessed on 24 August 2025). The calibration curve of the standard HA was analyzed using the linear regression analysis in Excel (Microsoft, 2016). For 16S rRNA diversity analysis, it was performed using the QIIME2 tool and R programming (version 3.6.0). DADA2 was used to obtain the final ASV feature list through filtering, dereplication, and chimera filtering operations [31]. PAST (PAlaeontological Statistics) software (version 4.03) was used for the rarefaction curves of sequence reads for the detected ASVs [32]. The principle coordinate analysis (PCoA) for beta diversity was visualized based on weighted and unweighted UniFrac distance estimations in R Studio (version 4.1.1, 2021-08-10) [33,34]. The heatmap was constructed using Heatmapper (http://www.heatmapper.ca, accessed on 24 August 2025) with Manhattan distance measurements and complete linkage hierarchical clustering between samples. The differentially abundant genera in diet and frass samples were analyzed using a row Wilcoxon-two-samples test using R Studio (version 4.1.1, 2021-08-10) with and adjustment of the *p*-value based on the Benjamini–Hochberg FDR method for multiple comparisons. An adjusted *p*-value < 0.05 was considered statistically significant.

## 3. Results

### 3.1. Larval Performance Across Feeding Substrates

Larval performance varied across the tested substrates (Table S1). The survival rate was consistently 100% across all diets, indicating that PBL were able to adapt well to the provided materials without adverse effects. However, the larval biomass increment was notably different among treatments. Larvae reared on diet A showed the highest biomass gain (0.15 ± 0.03 g/larva), which was more than double that observed for diets B and C (0.06 ± 0.03 and 0.06 ± 0.05 g/larva, respectively). The substrate reduction efficiency also reflected this trend, with diet A supporting the greatest substrate reduction (65.59 ± 2.42%), followed by diet B (62.33 ± 3.3%) and diet C (60.53 ± 0.05%). The bioconversion rate, which links larval biomass gain to substrate consumption, was likewise highest for diet A (21.22 ± 0.52%), while diets B and C yielded lower but comparable values (21.25 ± 0.43% and 19.90 ± 0.74%, respectively). These results suggest that the nutritional quality and degradability of diet A provided more favorable conditions for larval growth and conversion efficiency.

### 3.2. Phytotoxicity Assay of PBL Frass on Tomato

**Figure 1:**
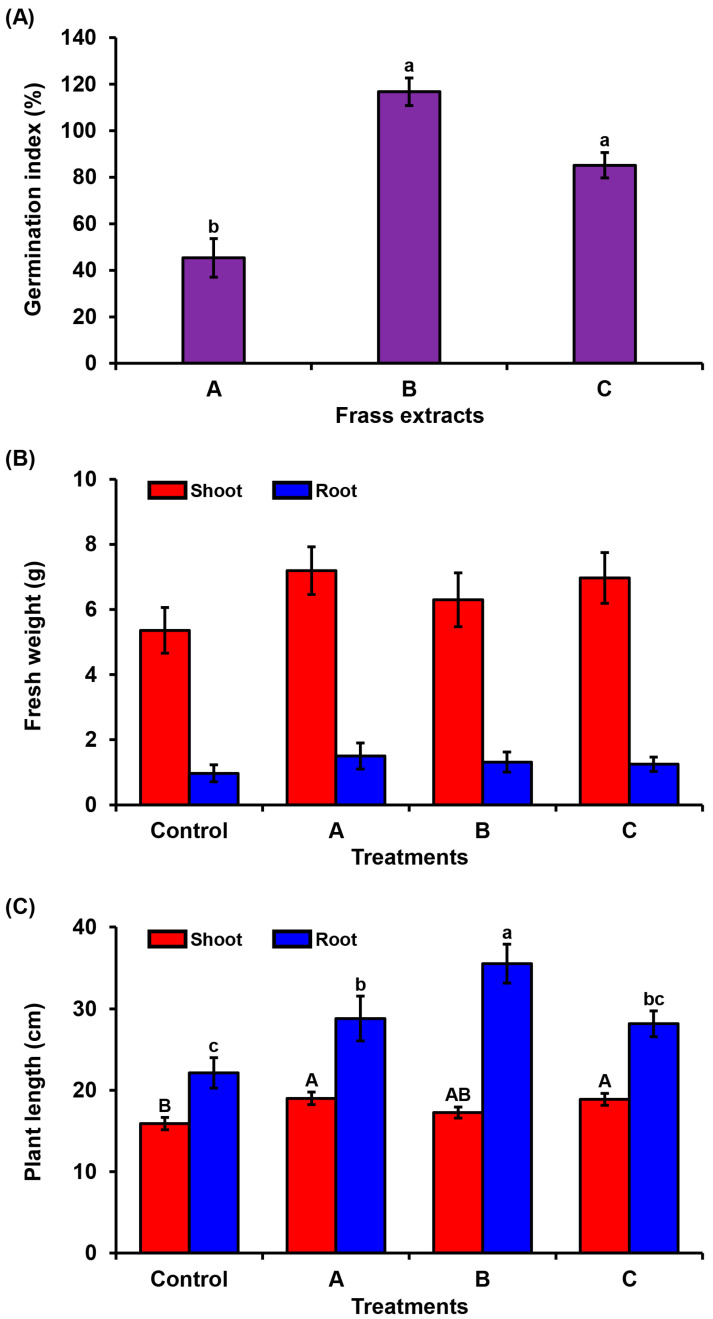
Effects of PBL frass on seed germination and plant growth. (**A**) The germination index of tomato seeds after treatment with the different frass extracts, A, B, and C. The bars represent the mean values ± standard error, *n* = 3. One-way ANOVA: *F*_2,6_ = 6.57, *p* < 0.05. (**B**) Fresh weights of shoot and root, mean values ± standard error, *n* = 5. One-way ANOVA: *F*_3,16_ = 1.17, *p* > 0.05 and *F*_3,16_ = 0.52, *p* > 0.05, respectively. (**C**) Lengths of shoot and root. The bars represent the mean values ± standard error, *n* = 5. One-way ANOVA: *F*_3,16_ = 4.01, *p* < 0.05 and *F*_3,16_ = 6.26, *p* < 0.01, respectively. The different uppercase letters on the error bars indicate significant differences between different shoot lengths, while lowercase letters indicate significant differences between different root lengths for each frass treatment based on a post-hoc LSD test (*p* < 0.05).

The phytotoxicity assay was performed using the tomato seed GI % and plant growth. The frass pH values recorded before and after incubation were for frass A (7.48 and 7.35, respectively), frass B (6.49 and 6.43, respectively), and frass C (5.68 and 5.56, respectively), indicating a slight change during the incubation time. As is shown in Figure 1, all frass extracts exhibited a GI value greater than 50%, indicating the absence of phytotoxic effects. Among the treatments, frass B demonstrated the highest GI value (104.27%), suggesting a potential stimulatory effect on seed germination. This was followed by frass C (97.92%), which also showed a strong positive response, and frass A (65.29%) which, while above the phytotoxicity threshold, exhibited a comparatively lower response (Figure 1A). This result suggests that all PBL frass samples have low phytotoxicity towards tomato germination with varying activity levels among frass types.

The plant growth assay not only showed no inhibition of tomato growth, but also, a noticeable increase in weight and length were found after the treatment of frass extracts (Figure 1B,C). The fresh weights of the tomato shoot and root indicated a notable increase in the treatment of frass A, B, and C (34.27% and 54.6%, 17.57% and 35.1%, and 29.99% and 28.9%, respectively), though not statistically significant (Figure 1B). Thus, the highest increase rate in the shoot and root weights was found in frass A (34.27% and 54.6%, respectively). The length of the shoot and root also indicated a significant increase in tomato plants, frass A (19.51% and 30.2%), frass B (8.62% and 60.62%), and frass C (18.82% and 27.35%) (Figure 1C). The maximum increase rate in shoot length was found in frass A (19.51%), while the maximum increase rate in root length was recorded for frass B (60.62%). These results indicate that the application of PBL frass does not cause growth inhibition in tomato plants. Variations in growth promotion among frass A, B, and C suggest that the composition of frass, chemical, microbial, or both, affects its bioactivity on plants. Additionally, despite a GI below 100%, the frass had a positive effect on subsequent seedling growth, indicating low phytotoxicity and potential biostimulant effects.

### 3.3. PBL-Based Bioconversion Led to Increasing HA Quantity and Quality

**Figure 2:**
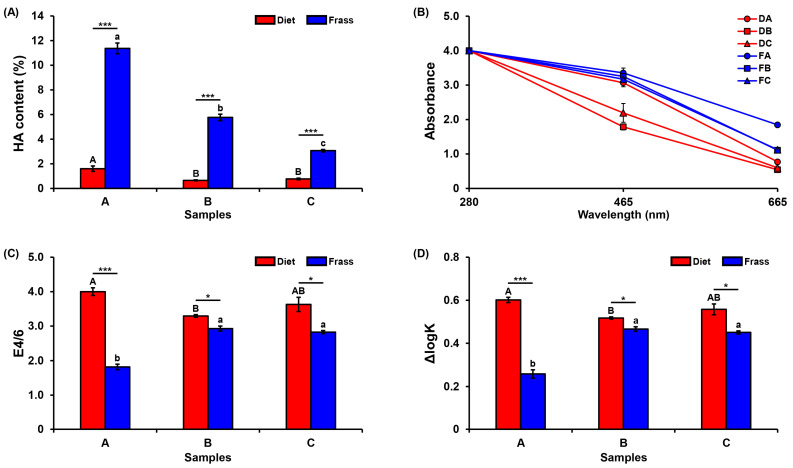
Humic acid quantification and qualification; (**A**) HA content (%), one-way ANOVA: *F*_2,6_ = 10.03, *p* < 0.05 and *F*_2,6_ = 133.31, *p* < 0.001, respectively. (**B**) Comparison of humic acids extracted from diet and frass samples in terms of the UV-spectrophotometer. Sample ID: DA, diet A; DB, diet B; DC, diet C; FA, frass A; FB, frass B; FC, frass C; (**C**) the humification index (E4/6 = absorbance at 465 and 665 nm), one-way ANOVA: *F*_2,6_ = 4.45, *p* > 0.05 and *F*_2,6_ = 53.73, *p* < 0.001, respectively. (**D**) ΔlogK (log E465 − log E665), one-way ANOVA: *F*_2,6_ = 4.47, *p* > 0.05 and *F*_2,6_ = 49.50, *p* < 0.001, respectively. The bars represent the mean values ± standard error, *n* = 3. The different uppercase letters on the error bars indicate significant differences between different diet samples, while lowercase letters indicate significant differences between different frass samples based on a post-hoc LSD test (*p* < 0.05). Asterisks on the error bars indicate a statistically significant difference (* *p* < 0.05, *** *p* < 0.001) between the diet and frass samples for each substrate (A, B, C), as determined by performing a *t*-test.

HA was extracted and calculated based on the dry weight of the HA yield divided by the dry weight of the source sample. Figure 2A demonstrates that the HA content was significantly increased in all frass samples in comparison to the diet samples. As is shown, frass A, B, and C increased to 11.37%, 5.77%, and 3.06%, respectively. These results demonstrate that PBL conversion led to a significant increase in the HA content of the resulting frass.

The calibration curve of standard-HA was generated, as demonstrated in Figure S1. The results based on the UV-spectrophotometer show a decrease in absorbance values when the wavelengths increase from 280 nm to 465 nm and 665 nm (Figure 2B). The ratio E4/6, which represents the HA absorbance at 465 nm and 665 nm, was significantly decreased in frass samples: A to 1.82, B to 2.93, and C to 2.82 (Figure 2C,D). The results demonstrated that all frass samples showed a significantly lower E4/6 ratio than diets, which reflects higher aromatic condensation and structurally mature HA, and frass A contains the most mature HA since it had the lowest E4/6 ratio (1.82) among all samples. This structural maturity is associated with slower mineralization, better soil organic matter persistence, and enhanced cation exchange capacity, making the frass-derived HA particularly valuable as a biofertilizer component.

### 3.4. Changes in Microbial Diversity and Composition Between Diet and Frass Due to PBL-Based Bioconversion

The diet and frass samples were analyzed using high-throughput 16S rRNA amplicon sequencing, which resulted in a total read count of 2,861,252, while 158,958 reads resulted on average per sample. Table S2 exhibits, in detail, some raw data metrics, including total bases, GC, AT, Q20, and Q30 percentages. After removing chimeric and mismatched sequences, the remaining sequences were clustered to 3458 ASVs.

#### 3.4.1. Changes in Microbial Diversity and Richness

The rarefaction curve demonstrates the sequencing depth of 16S rRNA amplicon reads, which were then clustered to ASVs. The total reads exceed 20,000 in all samples, while ASVs were noted to plateau at nearly 5000 reads, and their values were different throughout the samples (Figure S2).

**Figure 3:**
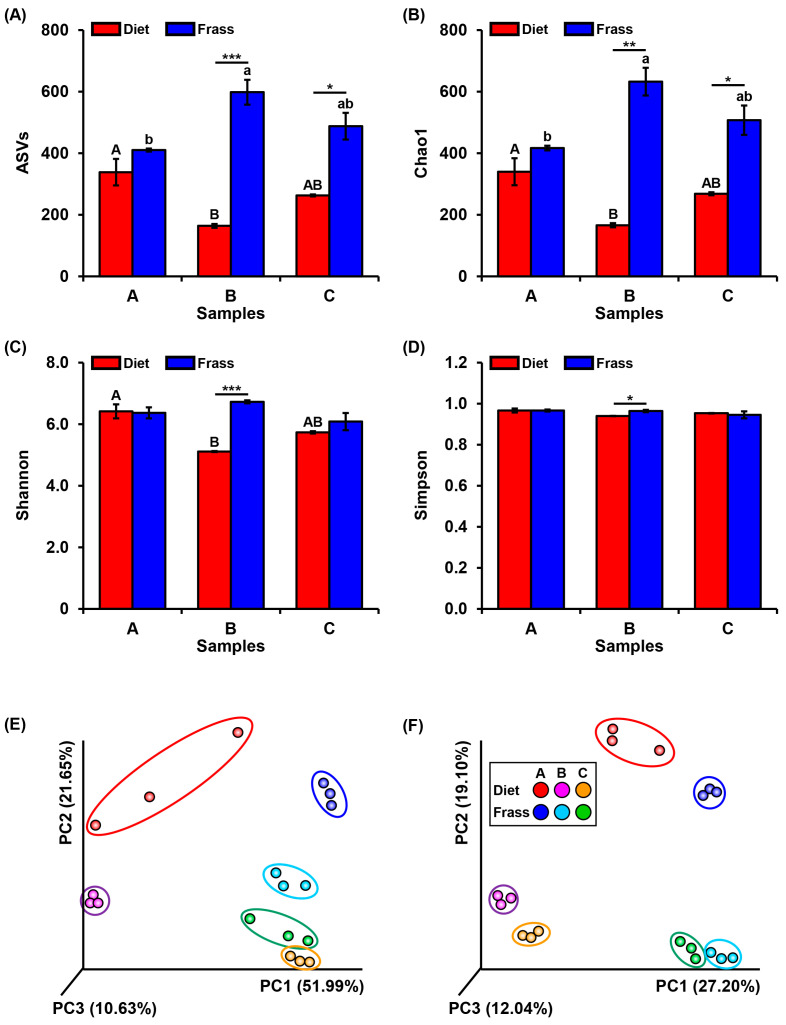
Alpha and beta diversity indices of the microbial community in diet and frass generated from high-throughput 16S amplicon sequencing. The alpha diversity indices, which measure the bacterial diversity within groups, include (**A**) the number of amplicon sequencing variants (ASVs), one-way ANOVA: *F*_2,6_ = 8.02, *p* < 0.05 and *F*_2,6_ = 5.02, *p* > 0.05, respectively. (**B**) Chao1 index, one-way ANOVA: *F*_2,6_ = 7.61, *p* < 0.05 and *F*_2,6_ = 5.38, *p* < 0.05, respectively. (**C**) Shannon index, one-way ANOVA: *F*_2,6_ = 15.84, *p* < 0.01 and *F*_2,6_ = 1.86, *p* > 0.05, respectively. (**D**) Gini–Simpson index, one-way ANOVA: *F*_2,6_ = 3.70, *p* > 0.05 and *F*_2,6_ = 0.73, *p* > 0.05, respectively. The bars represent the mean values ± standard error, *n* = 3. The different uppercase letters on the error bars indicate significant differences between different diet samples, while the lowercase letters indicate significant differences between different frass samples according to post-hoc Tukey’s test at *p* ≤ 0.05. The asterisks on the error bar represent significant differences between diet and frass samples for each substrate (A, B, C) according to a *t*-test: * *p* < 0.05, ** *p* < 0.01, *** *p* < 0.001. Principal coordinate analysis (PCoA) presenting beta-diversity, which measures the bacterial diversity between the groups of feeding diets and insect frass. (**E**) Weighted UniFrac and (**F**) unweighted UniFrac distances. The distinct clustering of frass vs. diet indicates consistent community restructuring via PBL feeding.

The alpha diversity indices were analyzed to show the microbial diversity within samples. The diversity metrics (ASVs, Shannon, Chao1, and Simpson) consistently indicated increased richness and evenness in frass compared to diet samples (Figure 3). This suggests that larval digestion created a more diverse microbial environment, likely reflecting the selective enrichment of taxa capable of metabolizing recalcitrant organic residues. For ASV numbers, there was a significant difference between diet B and frass B and between diet C and frass C, but no significant difference between diet A and frass A, which implies that the microbial community in these substrates remained relatively stable through larval digestion. It also revealed a significant difference between diets A and B and between frass A and B samples, in contrast to diet C samples and frass C as well (Figure 3A). Figure 3B exhibits the same pattern for the Chao1 index. The Shannon index showed a significant difference between diet B and frass B (*p* < 0.05) and between diets A and B samples; no other pairwise differences were detected (Figure 3C). In the Simpson index, there was a statistically significant difference only between diet B and frass B samples (Figure 3D). These results indicate that the microbial diversity and evenness were significantly altered in diet A, diet B, and frass B samples, while the microbial profile was more consistent in the other samples.

The beta diversity between samples was shown in a PCoA plot based on weighted and unweighted Unifrac distances. The PCoA plot exhibited a clear separation between all sample groups for both weighted and unweighted Unifrac analyses (Figure 3E,F), confirming that distinct microbial communities are present in each diet and frass. However, the sample groups of diet C, frass B, and frass C were closely clustered, more than the other groups, in the weighted Unifrac plotting (Figure 3E), indicating changes in dominant taxa due to larval digestion. In the unweighted Unifrac analysis, double-group close clustering was noted, including diet A and frass A, diet B and frass B, and diet C and frass C; the final was the closest (Figure 3F), suggesting that the presence or absence of taxa remains similar, even if relative abundances change. Taken together, these data provide insight into how the microbial community structure and diversity are influenced by the PBL digestion of different diet types. In addition, such clustering patterns indicate that PBL-mediated transformation not only alters substrate chemistry but also fosters microbial consortia with potential functional roles in humification and nutrient cycling.

#### 3.4.2. Changes in Microbial Composition

**Figure 4:**
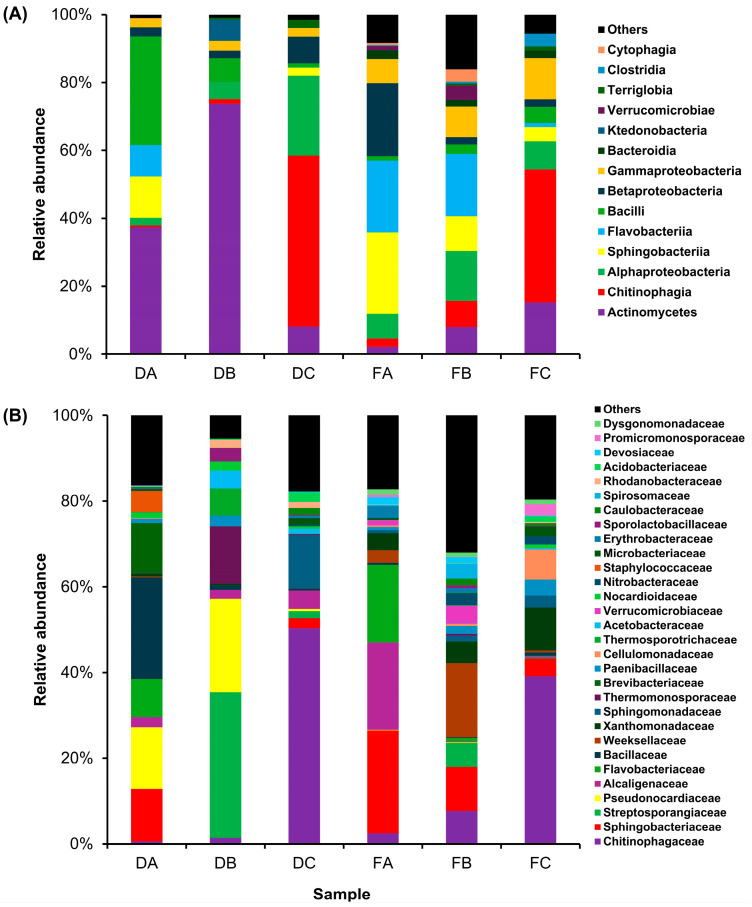
The microbial composition in PBL feeding diets and frass at (**A**) the class level and (**B**) the family level generated from high-throughput 16S rRNA amplicon sequencing. Sample IDs: DA, diet A; DB, diet b; DC, diet C; FA, frass A; FB, frass B; and FC, frass C. The bars represent the mean values, *n* = 3.

The microbial composition was noted to have remarkable differences among diet and frass samples. Figure 4 exhibits the relative abundances of the bacterial community at the class and family levels. At the class level, Actinomycetes, Chitinophagia, Alphaproteobacteria, and Sphingobacteriia were the most abundant within the whole community (24.10%, 16.93%, 10.15%, and 8.84%, respectively) (Figure 4A). Actinomycetes, which was the prominent class throughout the bacterial community, was found to have a variable distribution throughout the samples. In this regard, Actinomycetes was highly present in the samples of diet B, then diet A, and gradually decreased throughout the other samples until its lowest abundance in frass A samples. In contrast, Chitinophagia was the prominent class in the samples of diet C and frass C.

The relative abundance of the bacterial community at the family level is shown in Figure 4B. The five highly abundant families in the whole community were Chitinophagaceae, Sphingobacteriaceae, Streptosporangiaceae, Pseudonocardiaceae, and Alcaligenaceae (16.93%, 8.84%, 6.91%, 6.16%, and 4.96%, respectively). Chitinophagaceae was noted to be highly present in the samples of diet C and frass C, as well, while Sphingobacteriaceae had a noticeable presence in diet A, frass A, and frass B samples. The two families, Streptosporangiaceae and Pseudonocardiaceae, which belong to the Actinomycetes group, had a significant presence in diet B samples, while only Pseudonocardiaceae was noted in diet A samples.

**Figure 5:**
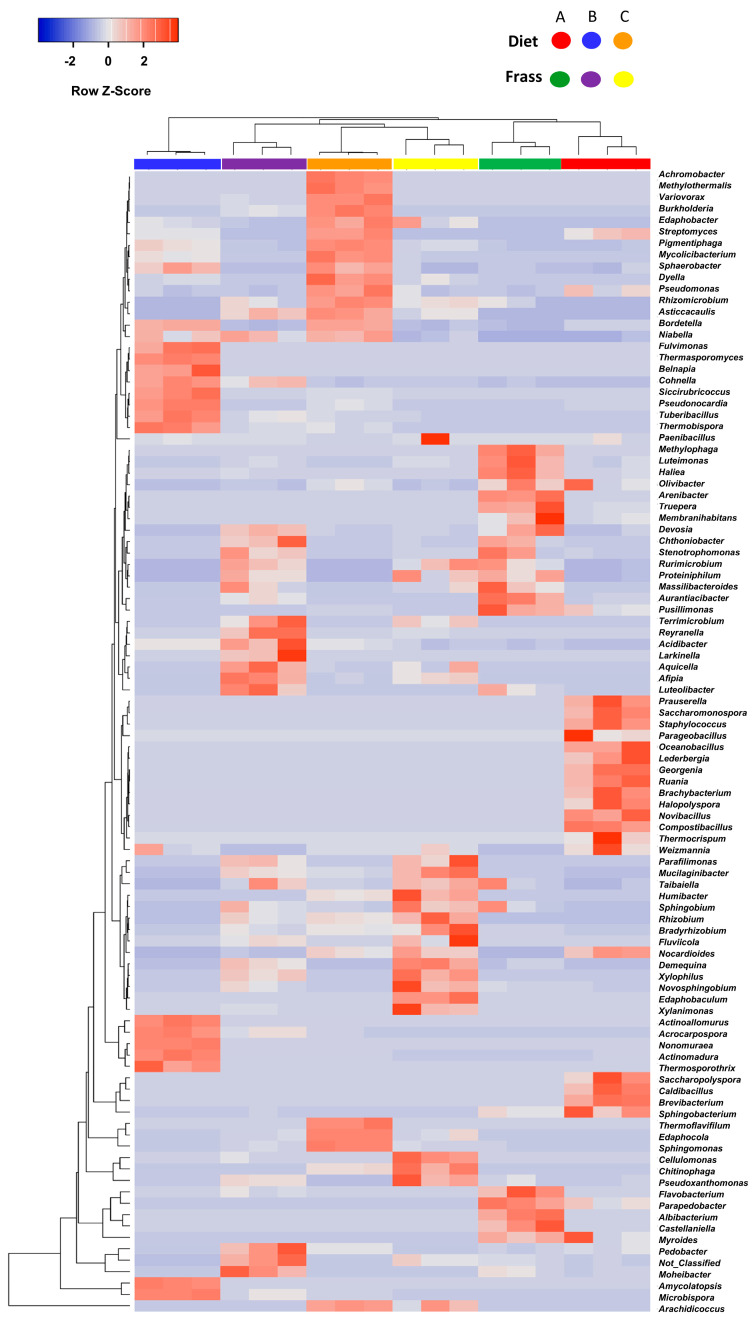
Heatmap based on the Manhattan distance measurement and the complete linkage clustering of the top 100 most abundant bacterial genera in the feeding diets and insect frass. The Z-Score represents the relative abundance of the bacterial genera in each row after heatmap standardization.

The variance of the bacterial composition between diet and frass samples was further measured based on a heatmap constructed according to the Manhattan distance with the complete hierarchical distance for the top 100 prominent genera in the whole community (Figure 5). The heatmap demonstrated that the samples of each diet and frass group were separately clustered together, indicating a significant difference in the microbial composition.

**Figure 6:**
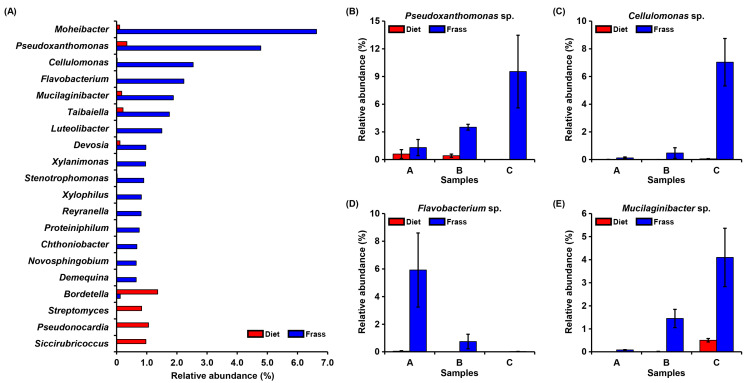
The different abundances of potential PGP genera in diet and frass samples. (**A**) The top 20 differently abundant genera between diet and frass groups. Bars indicate mean values (*n* = 9) of each bacterial genus’s relative abundance with significant differences (*p* < 0.05). The *p*-values < 0.05 were obtained after adjusting based on the Benjamini–Hochberg FDR method for multiple comparisons. The four abundant genera (ranked 2–5) were then selected for further analysis because they are previously reported as PGP microbes. (**B**–**E**) The relative abundances of the genera: *Pseudoxanthomonas*, *Cellulomonas*, *Flavobacterium*, and *Mucilaginibacter*. The bars represent the mean ± standard deviation, *n* = 3.

The differentially abundant genera in the PBL diet and frass group samples were determined based on the statistically significant difference (*p* < 0.05) and exhibited in Figure 6. Within top 20 genera, 16 genera (80%) had a higher abundance in frass than diet samples, including, for instance, *Mohibacter*, *Pseudoxanthomonas*, *Cellulomonas*, *Flavobacterium*, and *Mucilaginibacter*. These results highlight the selective enrichment of specific bacterial genera during the PBL-based bioconversion, indicating that larval digestion alters the microbial community composition in a way that may enhance the functional potential of the resulting frass.

**Figure 7:**
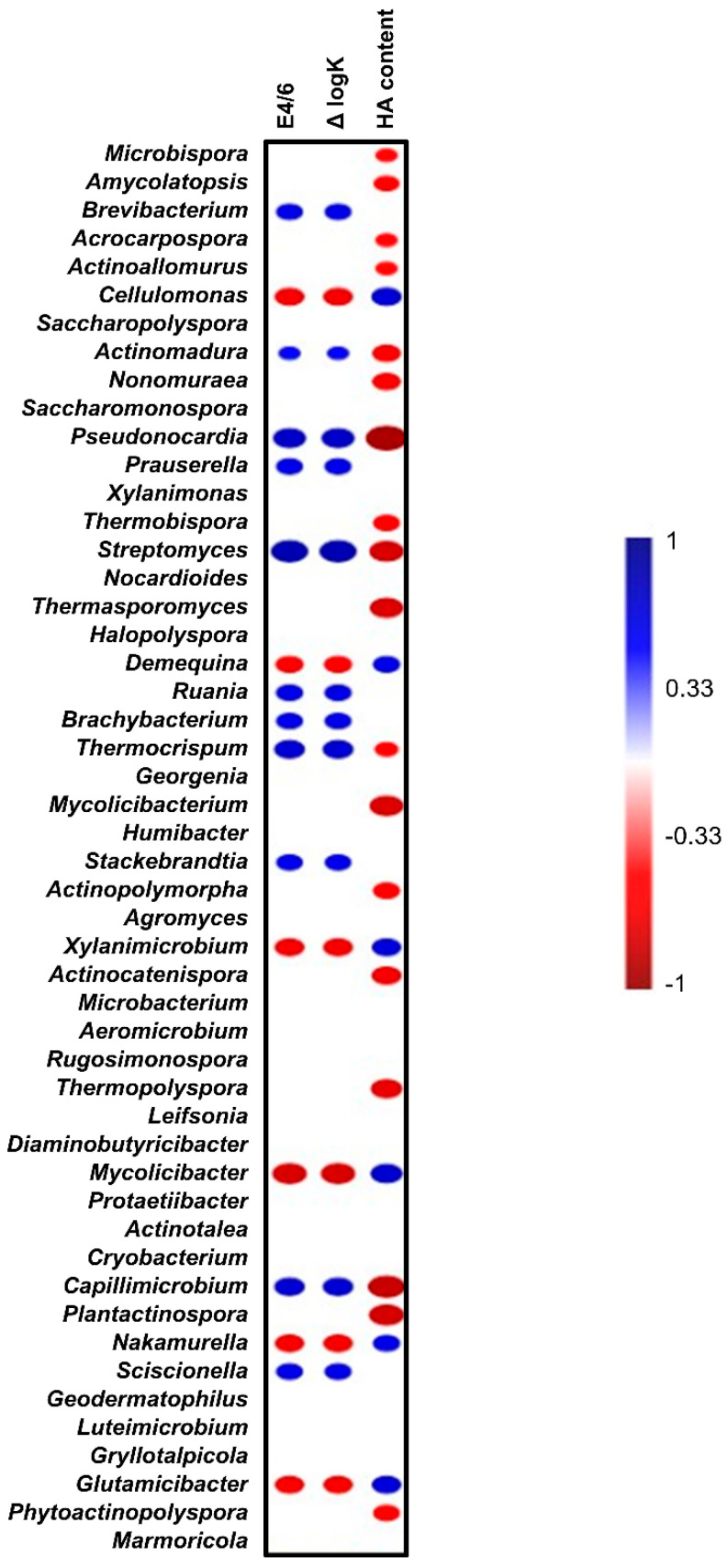
Correlogram illustrating Spearman’s rank order correlation coefficient (r) between the most abundant Actino-genera and the HA content and the humification indices, as well as E4/6 and ΔlogK, in diet and frass samples. Only significant (*p* < 0.05) positive (blue) and negative (red) correlations are shown in the graph (*n* = 18).

The correlogram shown in Figure 7 demonstrates the Spearman’s rank order correlation coefficient (r) between the top 50 Actino-genera and HA contents and the humification indices, as well as E4/6 and ΔlogK, in the whole set of diet and frass samples. Among all analyzed genera, there was a significant positive correlation between the abundances of six genera and (*n* = 18, *p* < 0.05) the HA content and the humification indices. These six genera included *Cellulomonas*, *Demequina*, *Xylanimicrobium*, *Mycolicibacter*, *Nakamurella*, and *Glutamicibacter*. This result suggests that these six genera are likely involved in or associated with the degradation and bioconversion of organic matter into humic substances during PBL digestion. While these findings suggest that PBL bioconversion may favor microbial taxa with beneficial functions, the observed patterns remain correlative and do not establish direct causality.

## 4. Discussion

PBL have shown potential in the bioconversion of agricultural residues, particularly lignocellulose-rich residues such as corn stalks, maize straw, and cottonseed hulls, into frass fertilizer enriched with HA and exhibiting low phytotoxicity [13,35]. However, the ability of PBL to convert *Pleurotus* SMSs and hardwood sawdust, both of which being challenging to degrade due to their complex lignin–cellulose composition, remains insufficiently explored. Specifically, the transformation efficiency of these substrates into frass with a high HA yield, desirable humification properties, and beneficial microbes has not been thoroughly characterized. A detailed analysis of the resulting frass, including a quantification of the HA content, evaluation of humification indices (E4/6 and ΔlogK), and profiling of the bacterial community structure, is essential to assess the quality and agronomic value of frass-fertilizer produced from these underutilized lignocellulosic wastes. Understanding these parameters will support the development of optimized insect-based waste-management strategies and contribute to sustainable organic fertilizer production.

In this study, frass derived from PBL rearing on agricultural byproducts exhibited low phytotoxicity, as all samples had germination index (GI) values above 50%. Importantly, none of the frass treatments inhibited tomato seedling growth; instead, they promoted root and shoot development. A GI value above 50% indicates the absence of major phytotoxins, while values exceeding 80% are considered indicative of full maturity and safety for agricultural use [36,37,38,39]. Given that SMS substrates are typically phytotoxic and unsuitable for direct applications [12], the marked improvement in the GI after larval bioconversion underscores the potential of PBL frass as a safe and plant-compatible biofertilizer. Although the observed differences in plant growth were below the threshold of statistical significance, they may still indicate subtle growth-promoting effects and low phytotoxicity, which are biologically relevant when evaluating frass as a potential biofertilizer [40,41]. Importantly, improved larval performance was generally associated with enhanced frass quality, as indicated by higher HA yields and more advanced humification in diet A frass. This aligns with earlier findings in *Hermetia illucens* and other saprophagous larvae, where rapid biomass accumulation and efficient substrate conversion were positively correlated with nutrient enrichment and organic matter stabilization in the residue [42]. Furthermore, a comparison of the C/N ratio revealed that diets A and B were similar, while diet C had a four-fold higher C/N ratio [43]. Thus, these parameters, including the food source C/N ratio and larval performance metrics, not only highlight the effectiveness of PBL for waste bioconversion but also provide mechanistic insights into how insect metabolism influences the chemical characteristics of the resulting frass. It could be concluded that PBL successfully converted SMS and sawdust residues to mature and phytotoxin-free frass. The difference between frass in plant growth may be due to the nutritional content that plays a role in plant growth promotion and alleviating toxicity effects [40].

Humic acids (HAs) are a chemical macromolecule with a flexible structure that differs based on the source, processing, environmental conditions, and presence of the functional groups. Due to HAs’ chemical properties, they have been known for decades for their applications in agriculture. For instance, HAs have a structural fraction range from neutral to acidic features that play a role in enhancing the soil pH [17]. HAs also play a unique role in plant growth, nutrient uptake, and crop yields at the end [16,44]. Additionally, HA is known to chelate micronutrients, making them more bioavailable to plants, and has been reported to modulate plant hormone-like effects that stimulate germination and root elongation [16,17]. By evaluating the HA content in our samples before and after PBL feeding, it was noted that the amount of HA is significantly increased after feeding. Therefore, the elevated HA content and maturity observed in this study supports the functional application of PBL-derived frass as a sustainable bio-fertilizer. These data confirm that PBL can convert agricultural residues to not only phytotoxin-free frass but also with a high amount of HA, which is compatible with the findings of [12].

In order to assess the quality of extracted HA, UV–spectrophotometry was used to record the absorbance at 280, 465, and 665 nm. As expected, the HA absorbance decreased with an increasing wavelength [15,45]. The E4/6 ratio, calculated as the absorbance at 465/665 nm, is widely recognized as an indicator of aromatic condensation, with lower values reflecting more mature and humified HA [46,47]. Beyond its chemical meaning, this structural maturity has strong agronomic relevance. Mature HA with low E4/6 ratios is more stable in soil, decomposes more slowly, and contributes to long-term soil organic matter persistence. Such HA also shows enhanced cation-exchange capacity, which improves nutrient retention and reduces leaching losses, while providing buffering capacity against pH fluctuations. Importantly, low E4/6 humic fractions have been shown to stimulate root elongation and nutrient uptake through hormone-like activities [16]. The practical benefits of these properties include improved fertilizer use efficiency, greater resilience of plants to abiotic stress, and enhanced soil health. Therefore, the significantly lower E4/6 ratios observed in frass-derived HA compared to diet-derived HA highlights their superior value as a biofertilizer component, with frass A being the most mature and agronomically promising sample.

Microbes have been reported to play vital roles in promoting the metabolism and development of the bioconverter insects during the bioconversion process [48]. The microbial analysis of diet and frass samples demonstrated a different bacterial structure in each group. On the class level, Actinomycetes was highly abundant in the whole community, and in the diet, it was higher than in frass samples, especially in diet A and B. This result is consistent with the investigation of [26], which reported a higher abundance of Actinomycetes in the midgut than in the hindgut. These findings may indicate that Actinomycetes main role is likely helping PBL in the digestion process in the midgut and is thus decreased in the hindgut and resulting frass. It may also confirm the hypothesis that insects mainly acquire microbes from diet and that those microbes are transmitted to the midgut where the favorable conditions of microbial activities exist [49,50]. On the other side, Actinomycetes have the ability to decompose lignocellulose by producing a variety of extracellular enzymes [51,52]. Therefore, Actinomycetes members seem to play a crucial role in structuring HA, which is usually generated from lignocellulosic substrates through a series of microbial reactions [53].

In this study, the top 20 significantly different microbes between diet and frass groups were investigated. It was noted that most of the bacterial genera have a higher abundance in frass than diet samples, and many genera have a positive impact on germination and plant growth. For instance, *Pseudoxanthomonas* was found to promote the growth of mung bean under stress conditions and cause a significant increase in the lengths of the plant shoot and root [54]. It also enhanced the plant ability of nitrogen fixation and the production of essential substances, including hydrogen cyanide and auxin. The inoculation of *Cellulomonas* strain, ZJW-6, in rice pots improved the available minerals and organic matter in soil and promoted plant growth [55]. In addition, the *Flavobacterium* genus has recently attracted the attention of researchers as potential beneficial microbes for plant cultivation [56,57]. Many functional properties of *Flavobacterium* are reported in plants, including disease resistance, abiotic stress tolerance, and growth promotion [58]. *Mucilaginibacter* has been reported to improve plant growth and salt resistance in several plants, such as *Arabidopsis* and maize crops [59,60]. These findings could indicate that PBL-based conversion led to an increase in the abundance of PGP microbes in the resulting frass. However, these findings should be considered correlative, and future work incorporating inoculation trials and metatranscriptomic or metabolomic approaches will be required to verify the causal relationships between frass-associated microbes and plant growth promotion.

The significant positive correlations observed between the abundance of specific actino-genera and the HA content, as well as humification indices (E4/6 and ΔlogK), suggest a potential functional role of these microbes in the transformation of organic residues into humic substances during PBL digestion. Notably, six genera, including *Cellulomonas*, *Demequina*, *Xylanimicrobium*, *Mycolicibacter*, *Nakamurella*, and *Glutamicibacter*, were consistently associated with higher HA yields and more advanced humification parameters. These genera are taxonomically diverse, yet are commonly reported in lignocellulolytic and composting environments, where they contribute to the breakdown of complex plant polymers such as cellulose, hemicellulose, and lignin [27]. The involvement of *Cellulomonas*, a well-known cellulolytic genus [55], further supports the hypothesis that the microbial degradation of polysaccharides is a key step in HA formation within the PBL gut or frass. The positive correlation between these taxa and ΔlogK, a parameter indicative of aromaticity and molecular complexity, suggests a possible microbial contribution to the structural maturation of HA during bioconversion. Although correlation does not confirm causation, the observed patterns strongly imply that these actinobacteria may play an active or associative role in humification processes during the larval digestion of agricultural residues.

## 5. Conclusions

This study evaluated the PBL efficiency in converting agricultural waste into frass fertilizer by characterizing the HA content and associated microbial communities. PBL successfully utilized SMS and sawdust substrates, producing phytotoxic-free frass enriched with HA and beneficial microbes. Microbial profiling indicated that genera such as *Pseudoxanthomonas*, *Cellulomonas*, *Flavobacterium*, and *Mucilaginibacter* may enhance frass efficacy in promoting plant growth and disease resistance. Significant correlations between the HA content and the abundances of actino-genera (*Cellulomonas*, *Demequina*, *Xylanimicrobium*, *Mycolicibacter*, *Nakamurella*, and *Glutamicibacter*) suggest their functional involvement in the humification process during PBL-mediated bioconversion. Future research should focus on isolating and characterizing these microbes to validate their roles as PGPs and bioindicators of effective organic matter transformation.

## Data Availability

The obtained sequences were deposited in the National Center for Biotechnology Information database as a sequence read archive under BioProject ID: PRJNA1071350.

## Supplementary Materials

The following supporting information can be downloaded at: https://www.mdpi.com/article/10.3390/insects16090893/s1, Figure S1: The calibration curve of standard–HA using a UV-spectrophotometer; Figure S2: Rarefaction curve of the microbial community generated from high-throughput 16S rRNA amplicon sequencing. Table S1. Survival rate, larval biomass increment, substrate reduction, and bioconversion rate of PBL on diet substrates; A, B, and C. Table S2: Results of high-throughput 16S rRNA amplicon sequencing representing total bases, reads, GC%, AT%, Q20%, and Q30% in diet and frass samples. Table S3. Amplicon sequence variants (ASVs) and alpha diversity parameters (Chao1, Shannon, and Gini–Simpson) of the microbial community.

## Abbreviations

The following abbreviations are used in this manuscript:

PBL	*Protaetia brevitarsis* larva
HA	Humic acid
LCB	Lignocellulosic biomass
SMSs	Spent mushroom substrates
PGP	Plant growth-promoting
