# Supplementary material for: Efficient Conversion of Mushroom and Sawdust Residues in Protaetia brevitarsis Biosystem: Characterization of Humic Acid and Bacterial Communities
Source: Insects. 2025 Aug 26;16(9):893. doi: 10.3390/insects16090893 (PMC12470708; doi:10.3390/insects16090893)
Supplement: Supplementary file 1 [file insects-16-00893-s001.zip › insects-3781442-supplementary.pdf]

Table S1. Survival rate, larval biomass increment, substrate reduction, and bioconversion rate of PBL on diet substrates; A, B, and C (mean ± standard error; n = 3).

Sample	Survival rate (%)	Larval biomass increment (g/larva)	Substrate reduction (%)	Bioconversion rate (%)
Diet A	100 ± 0.0	0.15 ± 0.03	65.59 ± 2.42	21.22 ± 0.52
Diet B	100 ± 0.0	0.06 ± 0.03	62.33 ± 3.3	21.25 ± 0.43
Diet C	100 ± 0.0	0.06 ± 0.05	60.53 ± 0.05	19.90 ± 0.74

* Larval biomass increment (g/larva) = [final total larval biomass (g) – initial total larval biomass (g)] / number of surviving larvae, substrate reduction (%) = [mass of feed at the start of the experiment (g) – mass of residue at the end of the experiment (g) / mass of feed at the start of the experiment (g) × 100%, bioconversion rate (%) = [total larval biomass (g) / feed added (g) × 100%.

Table S2. Results of high-throughput 16S rRNA amplicon sequencing representing total bases, reads, GC%, AT%, Q20% and Q30% in diet and frass samples.

Sample	Total bases(bp)	Total reads	GC (%)	AT (%)	Q20 (%)	Q30 (%)
Diet_A_1	39,957,148	132,748	56.9	43.1	89.4	79.4
Diet_A_2	33,439,294	111,094	54.6	45.4	90.3	80.8
Diet_A_3	45,963,302	152,702	56.2	43.8	90.2	80.7
Diet_B_1	41,993,112	139,512	59.3	40.7	87.8	77.3
Diet_B_2	53,998,798	179,398	59.3	40.7	87.3	76.8
Diet_B_3	54,167,960	179,960	59.1	40.9	88.8	78.7
Diet_C_1	50,208,606	166,806	53	47	91.8	83
Diet_C_2	58,307,312	193,712	53.1	46.9	92.1	83.4
Diet_C_3	52,852,590	175,590	53.3	46.7	91.7	82.9
Frass_A_1	27,853,938	92,538	52.9	47.1	91.7	82.8
Frass_A_2	41,489,840	137,840	52.6	47.4	92.3	83.8
Frass_A_3	48,754,174	161,974	52.6	47.4	91.5	82.4
Frass_B_1	57,919,624	192,424	53.9	46.1	91.6	82.7
Frass_B_2	60,322,206	200,406	53.5	46.5	91.4	82.5
Frass_B_3	53,034,996	176,196	54.3	45.7	91.3	82.2
Frass_C_1	51,595,012	171,412	53.9	46.1	91.2	82.1
Frass_C_2	49,245,406	163,606	52.6	47.4	92	83.3
Frass_C_3	40,133,534	133,334	52.2	47.8	91.9	83.2

Table S3. Amplicon sequence variants (ASVs) and alpha diversity parameters (Chao1, Shannon, and Gini-simpson) of the microbial community in feeding diets and insect frass generated from high-throughput 16s amplicon sequencing. (mean ± standard deviation; n = 3).

Sample	ASVs	Chao1	Shannon	Gini-Simpson
Diet A	338.67 ± 91.42	340.12 ± 93.26	6.42 ± 0.48	0.97 ± 0.02
Diet B	164.33 ± 13.05	166.25 ± 14.67	5.11 ± 0.03	0.94 ± 0.001
Diet C	263 ± 7.211	268.44 ± 10.64	5.74 ± 0.08	0.95 ± 0.002
Frass A	410.33 ± 10.26	416.92 ± 15.77	6.37 ± 0.38	0.97 ± 0.010
Frass B	598.33 ± 86.03	632.77 ± 95.69	6.73 ± 0.10	0.96 ± 0.01
Frass C	488 ± 92.15	507.65 ± 101.19	6.09 ± 0.59	0.95 ± 0.04

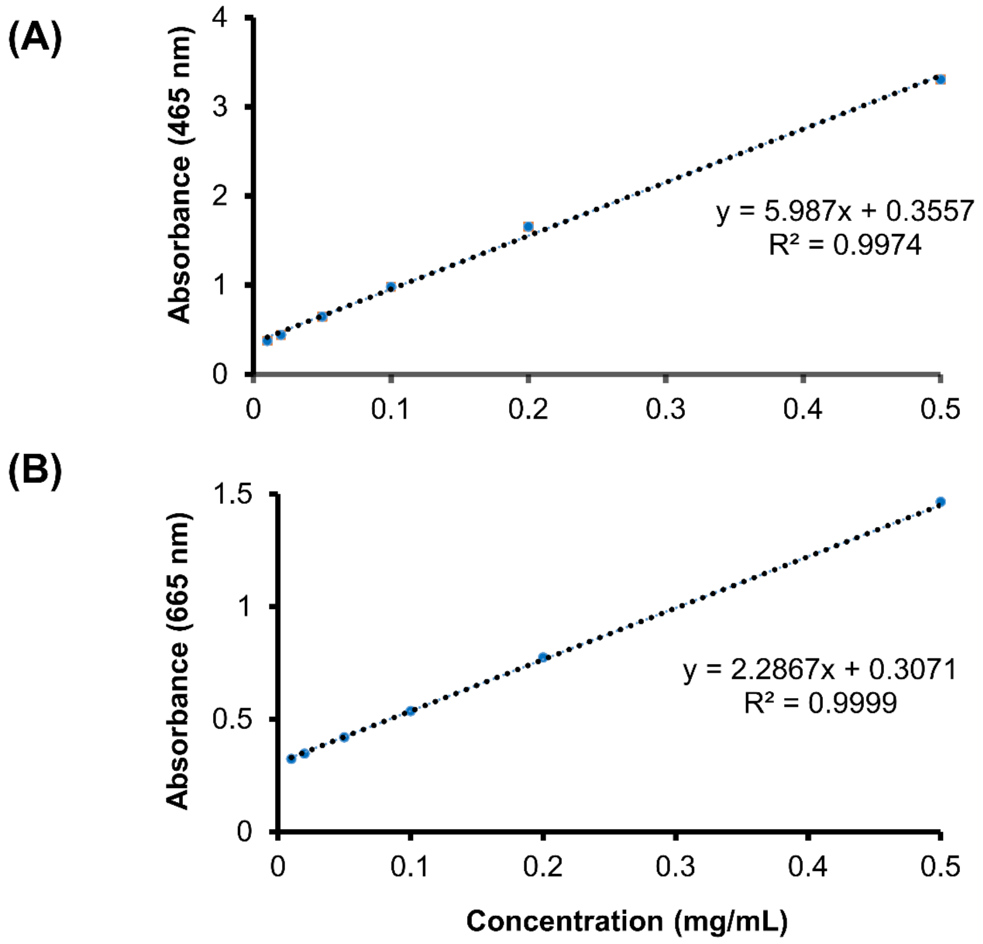

Figure S1. The calibration curve of standard-HA using UV-spectrophotometer. (A) The absorbance reads on 465 nm, (B) The absorbance reads on 665 nm. Several standard working solutions containing 0.01-0.5 mg/mL of HA was prepared, measured, and analyzed to get the regression formula and R^2 value which shown on the graph.

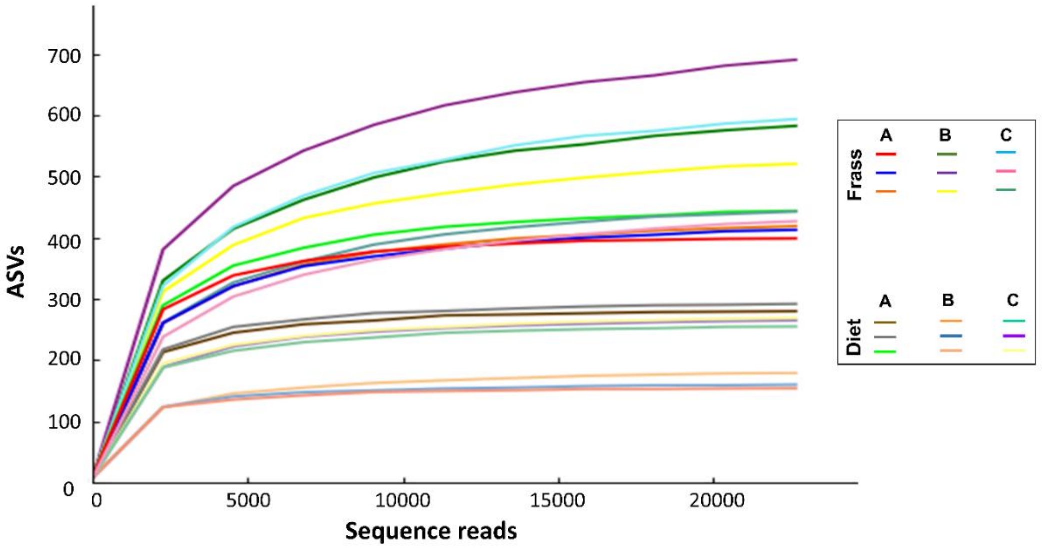

Figure S2. Rarefaction curve of the microbial community generated from high-throughput 16S rRNA amplicon sequencing. Rarefaction curve of the obtained 16S rRNA sequence reads against the assigned amplicon sequence variants (ASVs) for the sequence depth evaluation of diet and frass samples; A, B, and C.
